# Nephrotic syndrome after insect sting: a case report

**DOI:** 10.1590/2175-8239-JBN-2020-0014

**Published:** 2020-08-19

**Authors:** Vânia Junqueira, Beatriz Donato, Catarina Teixeira, Maria Isabel Mascarenhas, Isabel Costa Silva, Edgar Almeida

**Affiliations:** 1 Centro Hospitalar do Oeste, Departamento de Medicina Interna, Unidade de Caldas da Rainha, Caldas da Rainha, Portugal.; 2 Hospital Beatriz Ângelo, Departamento de Nefrologia, Loures, Portugal.; 3 Hospital Beatriz Ângelo, Departamento de Imunoalergologia, Loures, Portugal.

**Keywords:** Nephrotic syndrome, Nephrosis, Lipoid, Edema, Insect Bites and Stings, Pulmonary Embolism, Hypersensitivity, Síndrome nefrótica, Nefrose Lipoide, Edema, Mordeduras e Picadas de Insetos, Embolia Pulmonar, Hipersensibilidade

## Abstract

Minimal change disease accounts for up to 25% of the cases of nephrotic syndrome in adult population. The allergic mechanism has been associated with minimal change disease and allergens have been implied, namely insect stings. We present a case report of a woman with new onset of nephrotic syndrome after a non-hymenoptera insect sting, with biopsy-proven minimal change disease, that was accompanied by a pulmonary thromboembolism process. Complete remission with glucocorticoid therapy was observed, with sustained response for 6 months after discontinuation. A new exposure to insect sting in the same geographical region and season triggered a nephrotic syndrome relapse. Subsequent avoidance of the place resulted in a sustained remission for more than 4 years.

## RESUMO

A doença de lesões mínimas é responsável por até 25% dos casos de síndrome nefrótica na população adulta. O mecanismo alérgico tem sido associado à doença de lesão mínima a associada a alérgenos, como picadas de insetos. Apresentamos um caso de uma mulher com início recente de síndrome nefrótica após picada de inseto não himenóptero, com doença de lesões mínimas comprovada por biópsia, acompanhada por um processo de tromboembolismo pulmonar. A paciente teve remissão completa com glicocorticoides, com resposta sustentada por 6 meses após a interrupção do tratamento. Uma nova exposição à picada de inseto na mesma região geográfica e estação do ano provocou uma recaída da síndrome nefrótica. Evitar o local subsequentemente resultou em remissão sustentada por mais de 4 anos.

## INTRODUCTION

Nephrotic syndrome (NS) is characterized by nephrotic range proteinuria (>3.5 g/day in adults) associated with hypoalbuminemia, edema, hypercholesterolemia, and lipiduria. The common mechanism in NS is the loss of selectivity of the glomerular filtration barrier, allowing a massive flow of proteins into the urinary space.^1^ Venous thromboembolism is the most common complication of NS, most usually presenting with thrombosis of the deep veins of the lower limbs.^2^

Approximately 30% of adults with NS have an underlying systemic disease such as diabetes mellitus, amyloidosis, or systemic lupus erythematosus. The remaining cases are usually due to primary disorders including minimal change disease (MCD), focal segmental glomerulosclerosis (FSGS), and membranous nephropathy.^3^^,^ 4

MCD accounts for 10 to 25% of NS in adult patients,^4^^,^ 5 with a majority of them being idiopathic. Secondary causes of MCD include drugs such as nonsteroidal anti-inflammatory drugs, interferon α, lithium, or gold, and some hematologic malignancies.^2^

In the last decades, various reports suggested an association between NS and atopy. A history of allergy has been described in up to 30% of cases of MCD, with many allergens involved (pollens, mold, poison oak, dust, insect stings, and immunizations).^6^ The association of MCD with allergy and lymphoproliferative diseases, as well as the favorable response to steroids and other immunosuppressants, suggests a role of the immunological system.^1^

Increased serum immunoglobulin E (IgE) levels are commonly identified in atopic patients but may also be present in patients with idiopathic NS. High levels of interleukin 13, observed in NS, stimulate IgE response and may have a role mediating proteinuria in patients with MCD because of its ability to induce CD80 expression on the podocyte.^1^^,^ 6

In MCD there is no visible lesions at optical microscopy and neither deposits on immunofluorescence. The sole finding is the fusion of podocyte foot processes at electron microscopy.^1^

The association between the first episode or recurrence of NS with an insect sting has been previously reported in the literature, but only few cases have histological diagnosis. Case reports of MCD or FSGS have been described after hymenoptera (bee or wasp) or arachnid stings.^7^ Curiously, a case of NS by FSGS secondary to an ant sting has been described.^7^

We report a case of biopsy-proven MCD, with onset of NS after a non-hymenoptera insect sting and its recurrence after a new exposure to hypothetically the same insect in the same geographical area.

## CASE REPORT

A 35-year-old Caucasian woman went to the Portuguese southwest coast for camping during summer. One week later she noticed exuberant bilateral leg swelling and palpebral edema. She had no significant past medical history or known allergies and took no medication other than an oral contraceptive.

At hospital admission, she had generalized edema and a pustule with localized inflammatory signs on her right leg after an insect sting and weight of 63.5 kg (previously was 59 kg). She also presented a mild tachycardia (106 bpm), but otherwise normal physical examination, namely apyrexia and normal blood pressure.

Initial laboratory studies revealed: hemoglobin 15.7 g/dL, leukocytosis (15.100/ µL) with neutrophilia and eosinophilia (7.8%), C-reactive protein (CRP) 11.7 mg/dL, creatinine 0.57 mg/dL, hyponatremia (129 mmol/L), severe hypoalbuminemia (1.3 g/dL), hypercholesterolemia (total cholesterol 401 mg/dL and hypertriglyceridemia 240 mg/dL). The urinalysis showed proteinuria (++++) and rare red blood cells (1-5/field). Twenty-four-hour proteinuria was 22 g. Renal ultrasound was normal and the chest radiography presented bilateral pleural effusion.

The diagnosis of NS was established and immediate therapy with furosemide, angiotensin-converting enzyme inhibitor (ACEi), statin, and enoxaparin was initiated, as well as antibiotic therapy with amoxicillin and clavulanate.

Complementary investigation was inconclusive, namely negative antinuclear antibodies, negative antineutrophil cytoplasmic autoantibodies and normal complement levels. Serologies for HIV 1 and 2, HBV, and HCV were negative and serum protein electrophoresis excluded monoclonal gammopathy. Lower limbs venous Doppler ultrasound excluded deep venous thrombosis.

**Figure 1:**
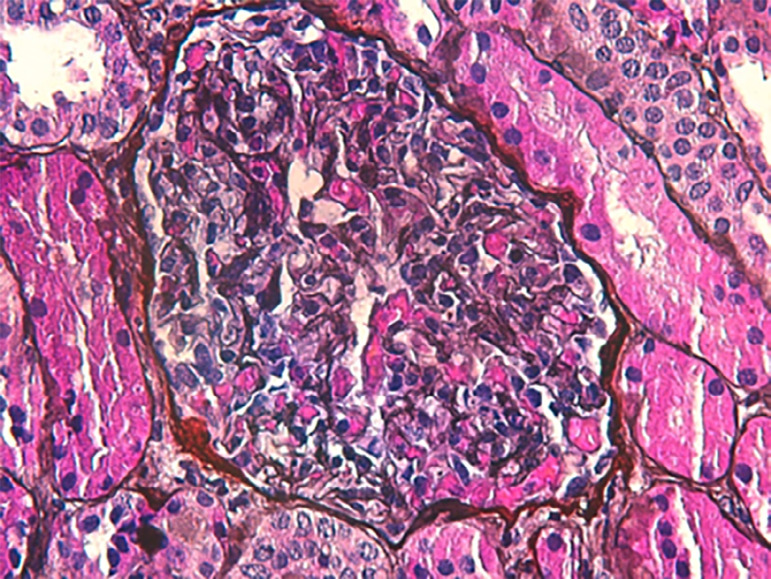
Renal biopsy featuring normal glomerulus and tubulointerstitium on light microscopy (silver 400x).

Renal biopsy was performed and showed normal glomeruli and tubulointerstitium on light microscopy, as well as absence of deposits on immunofluorescence, compatible with the diagnosis of MCD (Figure 1).

Prednisolone (1 mg/kg per day) was associated to the previous therapy and the patient was discharged 10 days later, with total resolution of pleural effusion and improvement of peripheral edema and inflammatory signs on the right leg. Three weeks after discharge, the patient was asymptomatic, with total resolution of the edema. Twenty-four-hour proteinuria was 207 mg and lipid profile normalized.

Prednisolone was slowly tapered during 6 months with a sustained complete remission.

One year after the first episode of NS, she went camping again in the same place. She started to notice progressive asthenia, followed by rapid development of edema and foamy urine.

Four days after the onset of symptoms, she was admitted in the emergency department and presented febrile (38ºC), with tachycardia (120 bpm) and a low blood pressure (99/56 mmHg). Severe peripheral edema and multiple pruriginous papular skin lesions caused by insect stings were observed in the thorax and lower limbs.

Admission laboratory tests showed a CRP 29 mg/dL, a normal renal function (creatinine 0.56 mg/dL) with normal urinary sediment and a proteinuria of 28 g/24 hours. Arterial blood gases and chest radiography were normal.

The patient was diagnosed with MCD relapse and corticosteroids were reinitiated, as well as furosemide, enoxaparin, and statin. Simultaneously, skin bacterial superinfection after insect sting was treated with antibiotic therapy. Phadiatop^®^ inhalant test was negative but serum total IgE was significantly elevated (>2000 UI/mL, with a reference level below 87 UI/mL). Specific IgE to common mosquitoes (*Aedes communis*) was positive (1.12 KU/L, reference values <0.10 KU/L). Circulating immune complexes were not assessed.

**Figure 2:**
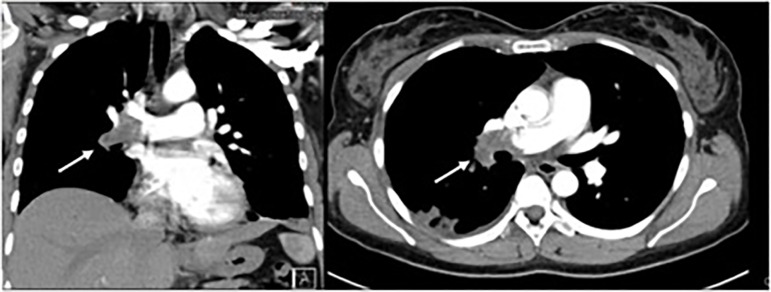
Chest computed tomography (CT): coronal section (left) and axial section (right) of central thrombus in the right pulmonary artery and main lobar branches.

On day 4 of hospitalization, she complained of sudden pleuritic chest pain on the right side, without other symptoms. Pulmonary examination, pulse oximetry, and arterial blood gases analyses were normal (PaO2 85 mmHg). Chest computed tomography disclosed complete occlusion of the middle lobe and lower lobe arteries of the right lung from the bifurcation of the right main pulmonary artery, translating extensive pulmonary thromboembolism process with areas of pulmonary infarction. The artery of the anterior basal segment of the left lower lobe also presented a luminal thrombus (Figure 2). She maintained anticoagulant therapy after discharge.

Five days after discharge, she was in complete remission of the NS (proteinuria 160 mg/24 hours). Corticotherapy was slowly tapered (1 year) and warfarin was maintained for 6 months. After the relapse episode, the patient decided to avoid the place where she used to spend holidays and she is still in complete remission since then (4 years).

## DISCUSSION

The association between insect stings and NS is rare and the evidence of a biopsy compatible with MCD in this setting is even less frequent.^8^ Fanconi et *al*, in 1951, were among the first who associated allergy and NS and since then various reports suggest a strong relationship between these two entities.^9^

The authors described a case of NS in a young woman, in which the diagnosis was easily established based on clinical and laboratory findings and MCD was confirmed by renal biopsy. Prompt glucocorticoid therapy was initiated with an excellent response, complete remission observed in a few days, and a sustained response even after therapy withdrawal.

One year later, the patient presented a NS relapse after exposure to the exact same circumstances observed in the first episode. That alerted us to the possibility of an underlying common triggering event for the development of NS. In both episodes the patient had been previously exposed to a non-hymenoptera insect sting in the same geographical area and season. In this scenario, it is reasonable to believe that she was stung by the same insect species both times, which led to NS. Causality between insect sting and NS development seems to be present in this case report, and becomes more evident when the avoidance of the conditions prevented relapse.

The presence of eosinophilia, high total serum IgE levels, presence of specific IgE antibody to the common mosquitoes, and the greater severity of the second episode also support an allergic/immunologic mechanism. The patient developed symptoms in a shorter period of time and presented with more severe skin lesion and tachycardia. Furthermore, fever and hypotension were absent in the first episode, but developed after the second exposure to the insect poison. We believe the faster and more severe reaction observed in the second episode led to an equally more severe presentation of NS with a life threatening extensive pulmonary thromboembolism despite prompt anticoagulation.

This report suggests the causality between insect sting and NS development, and an immune response could be responsible for the NS. An immunologic mediated response is supported by several aspects like the presence of eosinophilia and high IgE levels, presence of specific IgE antibody to the common mosquito, as well as the clinical presentation, namely the severity of the relapse and the sustained remission after avoidance of exposure, probably the longest described in literature.
